# Exploring mechanochemistry to turn organic bio-relevant molecules into metal-organic frameworks: a short review

**DOI:** 10.3762/bjoc.13.239

**Published:** 2017-11-14

**Authors:** Vânia André, Sílvia Quaresma, João Luís Ferreira da Silva, M Teresa Duarte

**Affiliations:** 1 Centro de Química Estrutural, Instituto Superior Técnico, Universidade de Lisboa, Av. Rovisco Pais, 1049-001 Lisbon, Portugal

**Keywords:** BioMOFs, drugs, green chemistry, mechanochemistry, organic based materials

## Abstract

Mechanochemistry is a powerful and environmentally friendly synthetic technique successfully employed in different fields of synthetic chemistry. Application spans from organic to inorganic chemistry including the synthesis of coordination compounds. Metal-organic frameworks (MOFs) are a class of compounds with numerous applications, from which we highlight herein their application in the pharmaceutical field (BioMOFs), whose importance has been growing and is now assuming a relevant and promising domain. The need to find cleaner, greener and more energy and material-efficient synthetic procedures led to the use of mechanochemistry into the synthesis of BioMOFs.

## Introduction

Mechanochemistry is a straightforward and clean technique by which the desired products are obtained in high purity and high or quantitative yield. It combines high reaction efficiency with a minimum input of energy and solvent. It is an approach to green chemistry, an area devoted to the discovery of environmentally friendly synthetic pathways, eliminating or drastically reducing the amount of solvent necessary to catalytically promote reactions. Mechanochemistry consists of grinding together two or more compounds to promote a reaction, by inducing the breaking/forming of covalent or supramolecular bonds [1–2].

There are different approaches towards mechanochemistry. The most direct is neat grinding (NG), in which the reagents are ground together without the addition of any solvent or other additive [3]. NG evolved into liquid-assisted grinding (LAG), also known as solvent-drop grinding or kneading, which includes the addition of catalytic amounts of solvent to facilitate the reaction. This technique proved to be useful for the synthesis of new compounds that could not be obtained by solution or NG techniques, while still avoiding excessive use of solvent [3–7]. The addition of catalytic amounts of an inorganic salt together with catalytic amounts of solvent, resulted in another mechanochemical approach, the ion and liquid-assisted grinding (ILAG), a technique that was also very successful in promoting solid-state reactions [8–11]. Polymer-assisted grinding (POLAG) is another variation of mechanochemistry, very recently disclosed and making use of polymers to stimulate the reaction [6,12].

Concerning the synthesis of molecular compounds and molecular crystals [2,13–15] mechanochemistry has been known for a long time [16–23] as a viable synthetic route and early works date back to the pioneer investigations by Etter [17–18,24], Rastogi [19,22–23] and Curtin and Paul [16,25–26]. Nowadays it is still a method of choice in different areas of chemistry and materials sciences, including organic solids [2] with pharmaceutical, luminescence- and thermoactive properties; studies of biomolecular recognition, asymmetric catalysis, interlocked systems and racemic resolution [2]. More recently mechanochemical methods were again successfully applied to the field of supramolecular chemistry [27–29], for solvent-free preparation of co-crystals, and adducts [30–38], polymorphs [12], supramolecular aggregates [4,30,39–42], host–guest complexes [5,43–45], metal-organic frameworks (MOFs) [8,28,44,46–50], and coordination networks [46–48,51].

All these applications comprise the formation of intermolecular interactions, the basis of supramolecular chemistry. This discipline was fully recognized internationally with the attribution of the Nobel Prize of Chemistry in 1987 to Donald J. Cram and Jean-Marie Lehn [52–55]. The energetics involved in supramolecular chemical reactions are not very severe, making mechanochemistry an excellent technique to be used in these processes.

In this short review, we particularly focused on the application of mechanochemistry to the synthesis of MOFs, especially on BioMOFs. MOFs are among the most exciting structures and their range of applications is rather vast, including, but is not limited to ion exchange, adsorption and gas storage [56–61], separation processes [62], catalysis [63–64], polymerization reactions [65–66], luminescence [67], non-linear optics [68] and magnetism [69], as well as contrast agents for magnetic resonance imaging (MRI) [70] and as drug carriers in systems for controlled drug delivery and release [64,71–80]. Also under development are new systems with potential use in further biomedical/pharmaceutical applications [71], such as cancer therapy [81–83].

MOFs combine coordination and supramolecular chemistry. Coordination chemistry is present in the coordination of organic molecules (linkers) to metal ions or clusters (coordination centers), while supramolecular chemistry relies on the formation of intermolecular interactions between linker molecules. This combination results in 1D, 2D or 3D porous frameworks. The pore size can be adjusted by varying the size of the linkers, a modification that can be associated to the change in functional groups in the organic moieties. These functional groups can form intermolecular interactions with potential pore incorporated molecules [72,84–86]. Their characteristics led researchers to explore the potential of MOFs as incarceration and/or delivery systems [70,79,83–87].

In BioMOFs, endogenous molecules, active pharmaceutical ingredients (APIs) or other bioactive organic molecules are used as building blocks for the framework [8]. Besides the advantages of MOFs as controlled delivery systems, BioMOFs have additional benefits, such as: i) porosity is no longer an issue as the release of the APIs or bioactive molecules is achieved by degradation of the framework, ii) no multistep synthesis is required as the molecules are part of the matrix itself, iii) synergetic effects between the active molecule and the metal may be explored, and iv) co-delivery of drugs is possible if a porous network is built with one ingredient and an incorporation of another is feasible [88]. BioMOFs are promising candidates for the development of more effective therapies with reduced side effects.

Two families of MOFs, MILs (materials of Institute Lavoisier) and CPOs (coordination polymers from Oslo), were the first to be studied for their potential medicinal applications. Here, the main focus was their use as drug-delivery systems [71–72,89], with particular attention to the toxicity of the metal centers [84]. Toxicity is a concern not only for the safe use of these compounds for humans but also for environmental reasons. These issues also led to the quest for biodegradable MOFs, the first being prepared in 2010 by Miller et al. [77].

Another family of MOFs, ZIFs (zeolitic imidazolate frameworks), that involves organic imidazoles as linkers, has been explored for medicinal purposes as a result of the enhancement of MOF structural and stability properties [90–91]. Bioactive molecules like caffeine [92–93] and anticancer drugs [94–98] were incorporated in ZIF-8 and tests proved that these systems allowed for a controlled drug release. Further studies involving ZIF-8 with encapsulated anticancer drugs have also shown that these have potential to be used in fluorescence imaging.

The number of reports on MOFs synthesized by mechanochemistry [8,28,50,99–101] has been increasing and some in situ studies on the mechanosynthesis of MOFs and coordination polymers are already being carried out with success. These studies show the propensity for stepwise mechanisms, especially in case of ZIFs, with a low density or a highly solvated product often formed first which is then transformed into increasingly dense, less solvated materials, resembling Ostwald’s rule of stages [8,102–107].

Many reviews on mechanochemistry [10,28–29,50,101,107–108] and MOFs [76,78–79,88,90,109] have been published due to the increasing relevance of both the technique and the type of compounds. We have recently published two reviews, one focused on the use of mechanochemical processes towards attaining metallopharmaceuticals, metallodrugs and MOFs synthesized within our group [49], and another on the design, screening, and characterization of BioMOFs in general [110]. To the best of our knowledge, this is the first short review targeting on the mechanochemical synthesis of BioMOFs.

## Review

### BioMOFs prepared by mechanochemistry and their main features

BioMOFs can be divided into two major classes: i) BioMOFs in which the APIs are the building blocks of the framework, thus excluding the need for large pores and ii) BioMOFs in which the API is incorporated (encapsulated) as a guest within the pores of the MOF. In the second situation, the choice of the linker is crucial, as it needs to be an organic molecule listed of the generally regarded as safe (GRAS) compounds, an endogenous compound or a bioactive molecule. In both classes, the judicious choice of the metals to be used in these systems is of great importance. Several metal species are known to display important biological activities that are applied for the treatment or diagnosis of several diseases. So, BioMOFs should contain either endogenous metal cations essential for life or exogenous metals that display a specific bioactive function in appropriate dosages, allowing to take benefits of possible synergetic effects between the metal and the APIs. Nevertheless, toxicity is also dependent on many other factors such as speciation, chemical nature, administration route, exposition time and accumulation/elimination from the body [88]. The examples given here will be separated according to the function of the APIs in the BioMOF: linker or guest.

### BioMOFs with active pharmaceutical ingredients (APIs) as linkers

**Figure 1:**
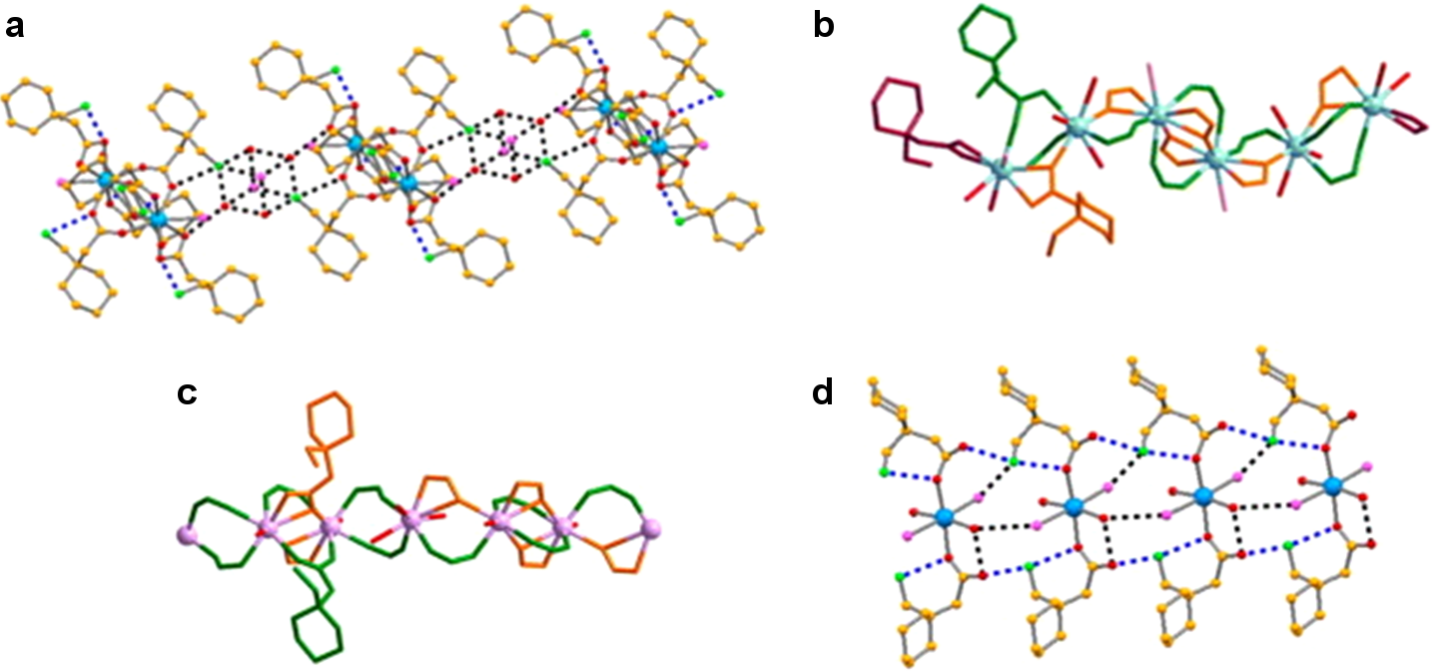
a) Detailed supramolecular packing of a gabapentin–Er network; b) view along the *b*-axis of the supramolecular packing of a gabapentin–Ce network; c) view of a GBP–Y network showing an infinite 1D chain; d) simplified packing of a gabapentin–Mn network. H atoms were omitted for clarity. Reprinted with permission from [49], copyright 2017 Elsevier.

Several BioMOFs with APIs as building blocks have been synthesized recurring to mechanochemistry and we will just present a few examples herein. It is common that these compounds are reported as coordination networks, or metallopharmaceuticals. One example we would like to mention has been proposed by Braga et al*.* [111], in which gabapentin was used as linker to build two new coordination complexes with ZnCl_2_ and CuCl_2_·2H_2_O by manually grinding both solids. Gabapentin is a neuroleptic drug used for the prevention of seizures, the treatment of mood disorders, anxiety, tardive dyskinesia [111–119], and neuropathic pain [120]. The synthesis of these coordination compounds with gabapentin was based on studies concerning the understanding of the physiological and pathophysiological roles played by Zn^2+^ and Cu^2+^ in various biological systems [121–123], and therefore the use of such coordination complexes was envisaged a new route for the delivery of those drugs. Gabapentin was also used by Quaresma et al. [124] in the synthesis by manual grinding of seventeen new metal coordination networks with Y(III), Mn(II) and several lanthanide chlorides (LnCl_3_), Ln = La^3+^, Ce^3+^, Nd^3+^, and Er^3+^. Ten out of these compounds were structurally characterized and represent the first coordination networks of pharmaceuticals involving lanthanides, showing different types of architectures based on mono-, di-, tri- and hexametallic centers and 1D polymeric chains. These new compounds proved to be unstable under shelf conditions. With regard to their thermal stability these compounds lose water at approximately 80 °C and melt/decompose above 200–250 °C [124]. This type of BioMOFs enclosing lanthanides and cations with potential luminescence properties can be explored for theranostic applications. Figure 1 shows some examples of the networks obtained.

Braga et al. synthesized new BioMOFs using 4-aminosalicylic acid and piracetam. 4-Aminosalicylic acid is an antibiotic that has been used in the treatment of tuberculosis, inflammatory bowel diseases, namely distal ulcerative colitis [125–126] and Crohn’s disease [127], while piracetam is a nootropic drug used to improve cognitive abilities. A 1D framework was synthesized which is stable up to 130 °C. The new compound resulting from the reaction between piracetam and Ni(NO_3_)_2_·6H_2_O consists of a polymeric chain based on a tetrameric repeating unit comprising a pair of piracetam molecules and two metal atoms and proved to be stable up to approximately 80 °C. Both BioMOFs were obtained recurring to manual mechanochemistry. Due to the possibility of synergic effects with Ag^+^, a known antimicrobial agent, the new network with 4-aminosalicylic acid and silver is highly interesting, as it represents a promising candidate to future biomedical applications [128].

**Figure 2:**
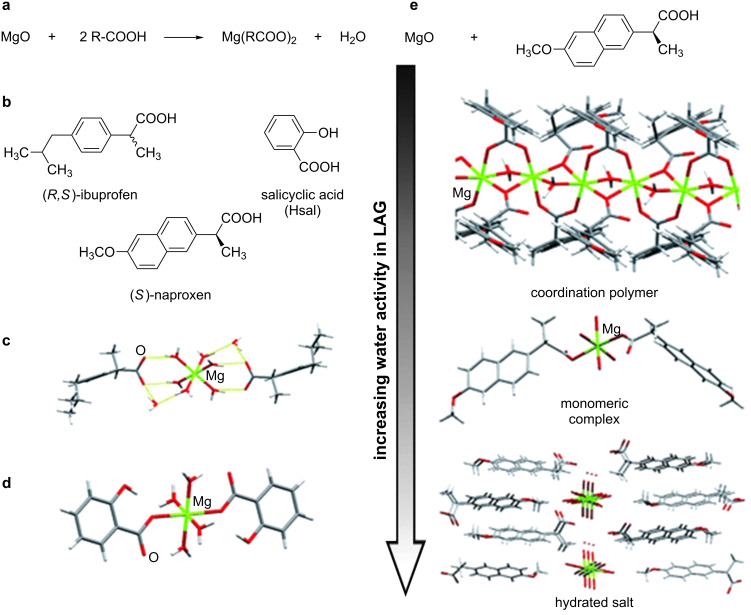
a) Mechanochemical reactivity between the excipient MgO and carboxylic acid NSAID molecules; b) NSAID molecules explored in mechanochemical reactions with MgO; c) fragment of the crystal structure of a mechanochemically obtained magnesium–ibuprofen complex; d) fragment of the crystal structure of a mechanochemically obtained magnesium–salicylate complex; e) screening for different hydrated forms of magnesium–naproxen BioMOFs by systematically varying the quantity of water in LAG reactions of MgO and (*S*)-naproxen. Reprinted with permission from [29], copyright 2012 the Royal Society of Chemistry.

Having in mind the synthesis of BioMOFs involving the excipient magnesium oxide initially proposed by Byrn et al*.* [129], Chow et al. and Friščić et al. developed new BioMOFs by LAG, grinding together MgO with the non-steroidal anti-inflammatory drugs (NSAIDS) ibuprofen (*S* and *RS*-forms), salicylic acid [130] and naproxen using water as the grinding liquid [7]. With naproxen, LAG was also used to screen for hydrated forms of magnesium–naproxen by systematically varying the fraction of water in the LAG experiments [7]. Low, intermediate and high amounts of water as grinding liquid led to the formation of a 1D coordination polymer monohydrate, a tetrahydrate complex and an octahydrate, respectively (Figure 2) [7,29].

### BioMOFs based on generally regarded as safe (GRAS), bioactive or endogenous linkers for the encapsulation of APIs

Another approach to build a BioMOF consists of the use of generally regarded as safe (GRAS), bioactive or endogenous linkers to form the 3D framework followed by the encapsulation of the APIs in the BioMOF. In these cases, the 3D frameworks may be synthesized by mechanochemistry, but the encapsulation of the drug is usually carried out by soaking methods. However, a significant number of these frameworks obtained by mechanochemistry with potential to be used as drug delivery systems have not yet been fully tested for the loading of drugs.

Pichon et al. proposed the first BioMOF synthesized by mechanochemistry using copper acetate and isonicotinic acid [46]. This type of compounds is useful for gas separation applications, but they haven’t been tested for biological applications yet. The solvothermal methods that were previously reported for the synthesis of this compound required high temperatures (150 °C), a 48 hours reaction and the use of solvents. With mechanochemistry, the same compound is obtained in high yield within 10 minutes at room temperature and without the use of solvents. Thus, this work revealed a fast, convenient, less expensive and effective preparative method for the synthesis of robust and stable 3D BioMOFs and rapidly inspired other groups to follow this methodology.

**Figure 3:**
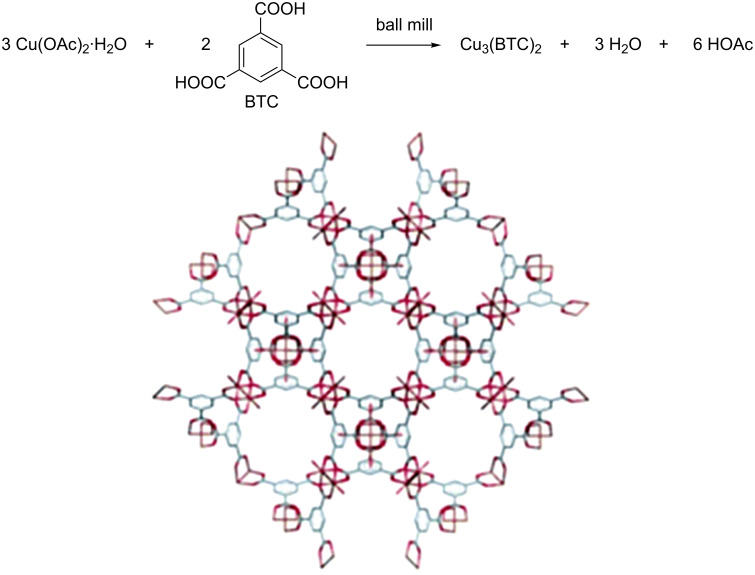
Mechanochemical reaction to form Cu_3_(BTC)_2_ and the structure of Cu_3_(BTC)_2_·(HKUST-1) as reported by Williams et al. [132]. Reprinted with permission from [131], copyright 2010 the Royal Society of Chemistry.

This has been proved by the work of Wenbing Yuan et al., in which a very important 3D BioMOF, known as HKUST-1, was synthesized by grinding together copper acetate with 1,3,5-benzenetricarboxylic acid (BTC, Figure 3) in a ball mill for 15 minutes without solvent. This procedure delivered HKUST-1 with some improved properties, including higher microporosity and surface area, when compared to those made in solution and by other techniques [131].

The presence of unsaturated open metal sites turns this compound into a potential adsorption/desorption material. Gravimetric tests with nitric oxide (NO), a gas with medicinal applications, demonstrated that HKUST-1, despite showing a reasonable aptitude to absorb this gas, displays very low rates of desorption when compared to others MOFs [56,84,133–134]. Furthermore, HKUST-1 is reported as a mean to achieve a controlled release of biologically active copper ions and it has shown to be an effective antifungal agent against representative yeast and mold [135].

**Figure 4:**
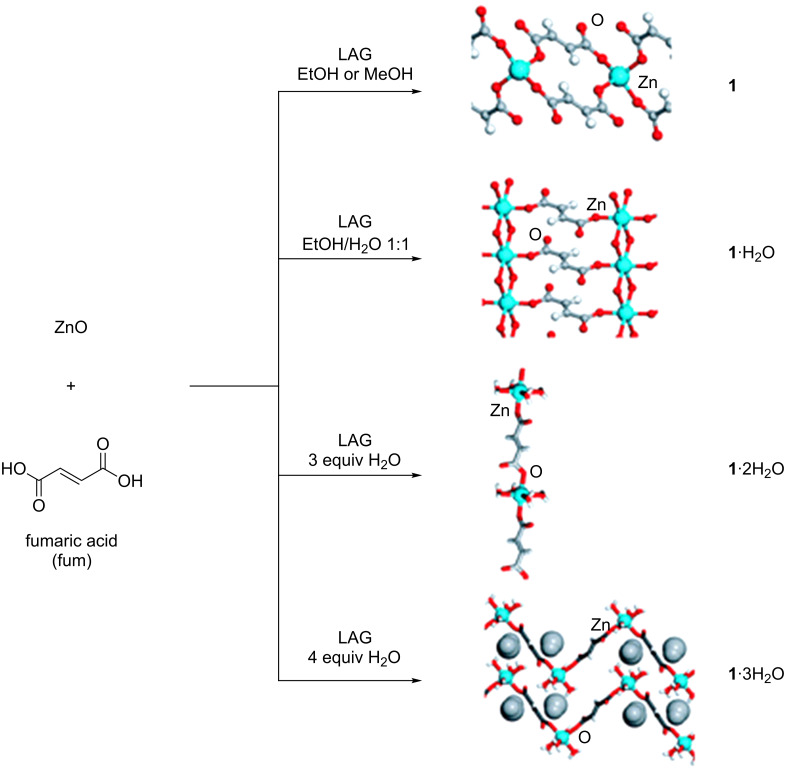
Mechanochemical syntheses of coordination polymers from ZnO and fumaric acid. Reprinted with permission from [137], copyright 2010 the Royal Society of Chemistry.

Friščić et al*.* also reported the synthesis of coordination polymers and BioMOFs using LAG by grinding together zinc oxide and fumaric acid. In this work, they initially obtained four different coordination polymers, depending on the choice of the grinding liquid: anhydrous zinc fumarate (**1**) when grinding with ethanol or methanol; a dihydrate (**1**·2H_2_O) when using a mixture of water and ethanol; a tetrahydrate (**1**·4H_2_O) and a pentahydrate (**1**·5H_2_O) when grinding with three or four equiv of water, respectively (Figure 4) [136–137].

**Figure 5:**
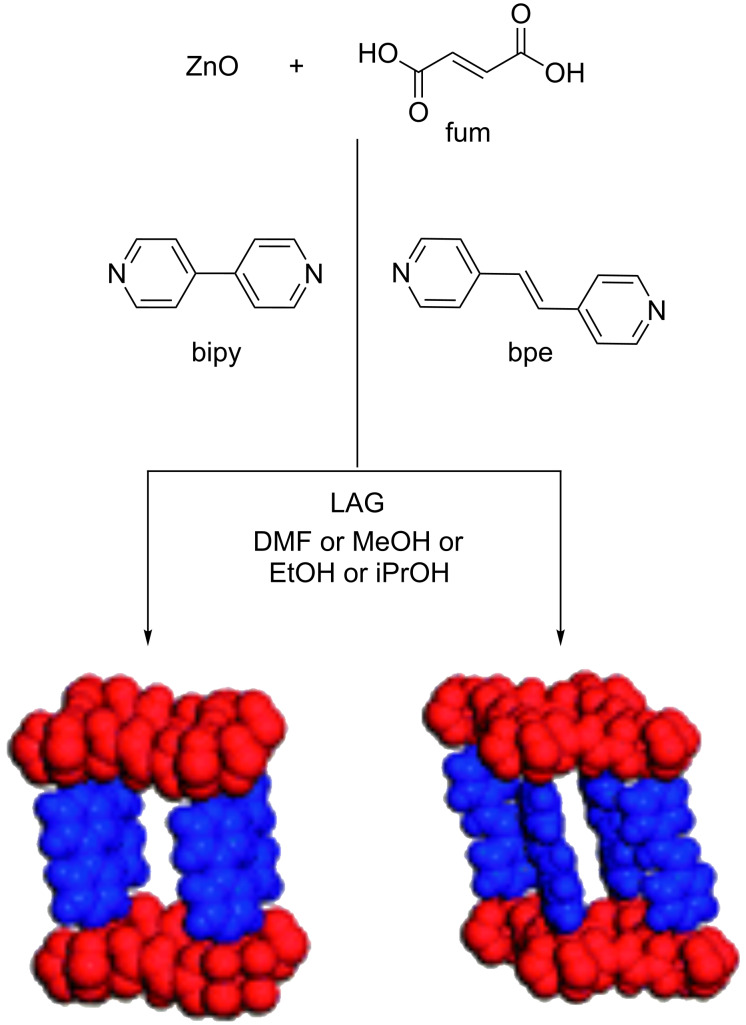
Mechanochemical synthesis of pillared MOFs from ZnO, fumaric acid and two auxiliary ligands (bipy and bpe). Reprinted with permission from [136], copyright 2009 the Royal Society of Chemistry.

This method was further applied to the mechanochemical synthesis of porous materials with introduced auxiliary ligands. These would allow for coordination to zinc in order to generate pillared MOFs, that could be used to incorporate APIs as a guest. Indeed, they synthesized two BioMOFs by grinding together zinc, fumaric acid and 4,4’-bipyridyl (bipy) or *trans*-1,2-di(4-pyridyl)ethylene (bpe) as ligands in the presence of a space-filling liquid agent (*N*,*N-*dimethylformamide, DMF). This synthesis also proceeded when using environmentally more friendly solvents, such as methanol, ethanol or 2-propanol, making these BioMOFs acceptable for biological and pharmaceuticals applications (Figure 5) [136,138]. However, studies supporting this goal have not been reported so far.

In 2015, Prochowicz et al. reported a new mechanochemical approach called “SMART” (secondary basic units-based mechanochemical approach for precursor transformation), in which pre-assembled secondary building units were explored. This method led to the successful synthesis of MOF-5 by mechanochemistry starting from Zn_4_O and 1,4-benzenodicarboxylic acid, without the need for bulky solvents, external bases or acids and high temperatures, all required in the conventional synthetic procedure [139].

Even though MOF-5 has not yet been tested for the incorporation of drugs, using the same linker, Xu et al. unveiled in 2016 the mechanochemical synthesis of MIL-101(Cr) involving heating which was successfully tested for the incorporation of ibuprofen. In this case, mechanochemistry proved once again to be a much faster process than the traditional hydrothermal synthesis that was used to obtain this compound involving solvents and often also hydrofluoric acid [140]. The linker used to build MIL-101 is 1,4-benzenedicarboxylic acid. Different applications of MIL-101 have been reported, from which we highlight the delivery of ibuprofen. MIL-101 exhibits a very high capacity of ibuprofen and therefore only very little quantities of MIL-101 are necessary for the administration of a high dosage of ibuprofen [141].

**Figure 6:**
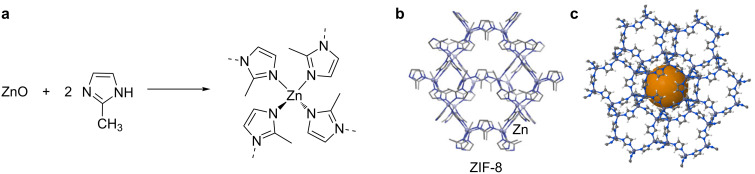
a) Synthesis of ZIF-8; b) fragment of the crystal structure of ZIF-8. Reprinted with permission from [145], copyright 2015 Macmillan Publishers Ltd. c) image generated for ZIF-8 in http://www.chemtube3d.com (University of Liverpool).

The mechanochemical synthesis was expanded by Beldon et al*.* to the synthesis of a very different family of metal-organic materials, the zeolitic imidazolate frameworks (ZIFs) [8]. ZIFs exploit a combination of metal ions and imidazolate linkers to build the 3D framework and have simultaneously the characteristics of MOFs and zeolites, making them very promising for biomedical applications [90–91]. In their work, Beldon et al. explored the synthesis of new ZIFs using imidazole (HIm), 2-methylimidazole (HMeIm) and 2-ethylimidazole (HEtIm) as ligands. Initially, they used LAG with ZnO and the previous imidazole ligands in the presence of DMF as a space-filling liquid. However, this method only partially succeeded: with HIm the quantitative formation of ZIF-4 was obtained after 60 min, whereas with HMeIm only partial formation of ZIF-8 was achieved and with HEtIm no reaction was observed at all [8]. As ILAG had already shown to accelerate and direct the formation of large-pore pillared MOFs [9], it was applied to these systems. A variety of ZIFs with defined topologies was obtained quantitatively by this method using ammonium nitrate, methanesulfonate or sulfate. Topology control could be achieved by either the solvent chosen for grinding or the choice of the salt additive. The most impressive result was the persistent formation of ZIF-8 (Figure 6) as it was obtained in all the reactions, showing the notable stability of this framework and making it a promising candidate to biomedical applications [8]. Indeed, ZIF-8 has been largely used to encapsulate APIs such as doxorubicin, an anticancer drug [96,142] or even as an efficient pH-sensitive drug-delivery system [92,95,143–144]. Usually, the encapsulation of small molecules into MOFs involves two steps: i) the synthesis of the framework and ii) the encapsulation of the small molecule by soaking and diffusion methods under mild conditions [96]. However, there are some one-pot syntheses reported for the encapsulation of small molecules into ZIF-8. Liédana et al. disclosed the in situ encapsulation of caffeine into ZIF-8 [98] and Zhuang et al. proposed a method to synthesize nanosized ZIF-8 spheres with encapsulation of small molecules into the framework during synthesis [95]. Also, Zheng et al. proposed a fast, single step synthesis of ZIF-8 with direct incorporation of small molecules, including doxorubicin [142]. The controlled drug release is due to the small pore size of ZIF-8 that prevents premature release and its pH sensitivity. At pH 5–6 there dissociation of the framework takes place with consequent drug release ideal to target cancer cells [95].

### Mechanochemistry in the synthesis of a metallodrug, another metal-organic target

**Figure 7:**
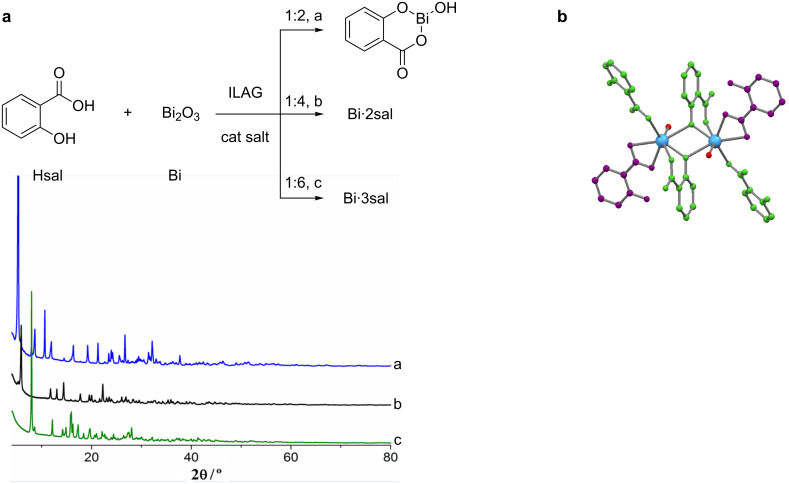
a) Mechanochemical reaction of salicylic acid with Bi_2_O_3_ yielding bismuth mono-, di- and trisalicylate, depending on the starting conditions; b) crystal structure of a bismuth disalicylate determined by XRPD data. Reprinted with permission from [149], copyright 2015 Wiley.

The study of the chemical reactivity of bismuth and carboxylic acids, in particular salicylic acid, is quite relevant for the pharmaceutical industry, because of the large production of bismuth subsalicylate (Pepto-Bismol), an anti-acid used in the treatment of stomach and intestine disorders. So far, this product was synthesized exclusively in solution involving harsh reaction conditions. André et al. [11] used ILAG [146–147] to prepare it directly from Bi_2_O_3_ (Bi) and salicylic acid (SA) in a 1:1 (Bi·SA) stoichiometry. This method proved not only to be more efficient but also very selective [11]. Changing the stoichiometric ratio of the reactants to 1:2 and 1:3 allowed the syntheses of another two bismuth–salicylate compounds, namely the disalicylate and the trisalicylate, respectively. The only previously known crystal structure obtained for bismuth salicylates was a Bi_38_ cluster isolated by recrystallization of the trisalicylate from acetone [148] and this was then considered a possible model for the structure of bismuth subsalicylate [11]. In 2011, André et al. performed a similar recrystallization of the disalicylate and obtained a similar Bi_38_ cluster with coordinated *N*,*N-*dimethylformamide (DMF) molecules instead of acetone, showing the structural robustness of this core in solution. The crystal structure solution from powder X-ray diffraction data of the disalicylate revealed the first crystal structure of a bismuth salicylate without coordinated solvent molecules (Figure 7). This indicates that bismuth salicylates form extended structures without the presence of other ligands [11].

## Conclusion

All examples presented herein and collected in Table 1 show the advantages of combining pharmaceutically relevant organic molecules with metal centers, in order to obtain compounds with enhanced biological properties.

**Table 1:** Summary of the BioMOFs synthesized by mechanochemistry presented herein.

Metal	Linker	Ref.

Zn^2+^, Cu^2+^	gabapentin	[111]
La^3+^, Ce^3+^, Nd^3+^,Er^3+^, y^3+^, Mn^2+^	gabapentin	[124]
Ag^+^	4-aminosalicylic acid	[128]
Ni^2+^	piracetam	[128]
Mg^2+^	ibuprofen, naproxen, salicylic acid	[7]
Cu^2+^	isonicotinic acid	[46]
Cu^2+^	1,3,5-benzenetricarboxylic acid	[131]
Zn^2+^	fumaric acid	[136]
Zn^2+^	fumaric acid + 4,4‘-bipyridine	[138]
Zn^2+^	fumaric acid + 1,2-di(4-pyridyl)ethylene	[138]
Zn_4_O	1,4-benzenedicarboxylic acid	[139]
Cr^3+^	1,4-benzenedicarboxylic acid	[140]
Zn^2+^	2-methylimidazole	[8]
Zn^2+^	2-ethylimidazole	[8]
Bi^3+^	salicylic acid	[11]

New metal-organic frameworks, BioMOFs, for the use of controlled drug delivery and/or release or other biological applications, were successfully synthesized either by direct incorporation of the bioactive molecule in the framework (linker), or by encapsulation (guest). Mechanochemistry has proved to be an efficient, high performance, environmentally friendly, cleaner, and faster synthetic procedure, leading to significantly lower costs of production.

There is still much to explore in the combination of BioMOFs with mechanochemistry and this is certainly an expanding area in the field of organic coordination chemistry.
